# *In Vitro* Silencing of *Brugia malayi* Trehalose-6-Phosphate Phosphatase Impairs Embryogenesis and *In Vivo* Development of Infective Larvae in Jirds

**DOI:** 10.1371/journal.pntd.0001770

**Published:** 2012-08-14

**Authors:** Susheela Kushwaha, Prashant Kumar Singh, Mohd. Shahab, Manisha Pathak, Shailja Misra Bhattacharya

**Affiliations:** Division of Parasitology, CSIR-Central Drug Research Institute, Lucknow, India; McGill University, Canada

## Abstract

**Background:**

The trehalose metabolic enzymes have been considered as potential targets for drug or vaccine in several organisms such as *Mycobacterium*, plant nematodes, insects and fungi due to crucial role of sugar trehalose in embryogenesis, glucose uptake and protection from stress. Trehalose-6-phosphate phosphatase (TPP) is one of the enzymes of trehalose biosynthesis that has not been reported in mammals. Silencing of *tpp* gene in *Caenorhabditis elegans* revealed an indispensable functional role of TPP in nematodes.

**Methodology and Principal Findings:**

In the present study, functional role of *B. malayi tpp* gene was investigated by siRNA mediated silencing which further validated this enzyme to be a putative antifilarial drug target. The silencing of *tpp* gene in adult female *B. malayi* brought about severe phenotypic deformities in the intrauterine stages such as distortion and embryonic development arrest. The motility of the parasites was significantly reduced and the microfilarial production as well as their *in vitro* release from the female worms was also drastically abridged. A majority of the microfilariae released in to the culture medium were found dead. *B. malayi* infective larvae which underwent *tpp* gene silencing showed 84.9% reduced adult worm establishment after inoculation into the peritoneal cavity of naïve jirds.

**Conclusions/Significance:**

The present findings suggest that *B. malayi* TPP plays an important role in the female worm embryogenesis, infectivity of the larvae and parasite viability. TPP enzyme of *B. malayi* therefore has the potential to be exploited as an antifilarial drug target.

## Author Summary

Lymphatic filariasis, one of the neglected tropical diseases, is the second leading cause of permanent and long term disability. Control of the disease relies on the mass administration of drugs which mainly act on the microfilariae without substantial effect on adult worms. Drugs need to be continued for several years to block the transmission of infection which may result in to development of resistant parasites. The sugar trehalose has been shown to play several important functions in the nematodes, and trehalose biosynthetic enzymes have been considered as potential targets for drug or vaccine candidate. In the present study we silenced trehalose-6-phosphate phosphatase and studied the biological function of TPP enzyme in the filarial nematode *B. malayi* viability, female worm embryogenesis and establishment of infection in the host. *In vitro* gene silencing was done in adult parasites using 5 mM concentration of siRNA while 2 mM of siRNA was used to treat L3 which were further inoculated into the peritoneal cavity of jirds to study the effect of siRNA treatment on *in vivo* larval development. The present findings validate trehalose-6-phosphate phosphatase as a vital antifilarial drug target.

## Introduction

Lymphatic filariasis (LF), caused by mosquito transmitted filarial parasites, *Wuchereria bancrofti, Brugia malayi and B. timori* has been identified as a second leading cause of permanent and long term disability. LF control relies on community-wide mass distribution of diethylcarbamazine or ivermectin in combination with albendazole [1]. These drugs are principally microfilaricidal and not much effective on the adult worms, thus repeated treatment is required over many years for interrupting the transmission. This also raises the possibility of parasites becoming resistant to these drugs. The indications of resistance are already being noticed in case of albendazole and ivermectin [2], [3]. The renewed efforts are needed to discover macrofilaricides and/or embryostatic agents in addition to new microfilaricides to address the emerging threat of resistance. One of the methods to achieve this goal is to identify and inhibit the genes or proteins that play a crucial role in the growth and the survival of filarial worms. Complete genome information of *B. malayi* [4], [5] now permits identification of the key enzymes and their pathways that can be specifically targeted. Reverse genetic studies such as RNA interference (RNAi) provides a more direct approach for identifying such genes/gene functions and offers a valuable tool for modern drug discovery. The trehalose metabolism pathway in the nematodes provides an attractive drug target sites. Sugar trehalose, a disaccharide of glucose, plays a significant role in the parasite biology such as, in egg hatching, as stress protectant, in glucose uptake and serving as an energy reservoir [6]–[9]. Trehalose biosynthesis pathways are widely distributed in nature, except in vertebrates. There are five known trehalose biosynthetic routes in prokaryotes: Trehalose-6-phosphate synthase (TPS)/trehalose-6-phosphate phosphatase (TPP), Trehalose synthase (TS), maltooligosyl-trehalose synthase (TreY)/maltooligosyl-trehalose trehalohydrolase (TreZ), Trehalose phosphorylase (TreP) and trehalose glycosyltransferring synthase (TreT). In the invertebrates, only the first pathway TPS/TPP is known to exist [10]. The synthesis of trehalose in the nematodes proceeds in the classical pathway and is catalysed by the action of two enzymes: i) TPS, which catalyses the transfer of glucose from uridine diphosphate (UDP)-glucose to glucose-6-phosphate to produce trehalose-6-phosphate (T6P); and ii) TPP, which converts T6P to free trehalose and Pi [11]. Both the enzymes represent a set of attractive drug targets as no homologues are present in the mammals [12]. We earlier reported on the cloning, expression and purification of *B. malayi* TPP that was found to be an unusual phosphatase [13]. In the nematodes, TPP was first identified in *C. elegans* in a forward genetic screen for intestinal defects where the loss of *tpp* function resulted in to early larval lethality due to blocked intestinal lumen and consequent starvation [14], [13]. TPP is also considered to be a potential drug target in *Mycobacterium* due to its role in the cell wall biosynthesis [15]. In the present study, we demonstrate for the first time the biological function of *tpp* in filarial parasite, *B. malayi* by *in vitro* RNAi mediated gene silencing and validated it as a putative antifilarial drug target.

## Materials and Methods

### Synthesis of siRNA

The gene specific siRNA for *B. malayi tpp* used in the present study were custom designed and synthesized by Ambion (USA). The highest ranking sense and anti-sense siRNA duplexes representing the best combination of activity and specificity were provided with a concentration of 40 nmoles as lyophilized powder. 100 µM stock solution was prepared and stored at −20°C. The sequences of the sense and antisense strands of siRNA are:

Sense 5′GGA UGA AGG UUU CAA CGC Att′3

Antisense 5′ UGC GUU GAA ACC UUC AUC Cgt′3

siRNA (#AM 4621, Ambion) completely unrelated to *B. malayi* that does not target any gene product was used as a negative control to determine off target effects, if any. The negative control siRNA does not have any sequence similarity to mouse, rat or human gene sequences and have been pretested (Ambion) in cell based screens and proven to have no significant effect on cell proliferation, viability or morphology.

### Animals

Purpose-bred, parasite naive, six week old, male, jirds (*Meriones unguiculatus*) were used in the study. The animals were maintained in proper housing condition in the Animal House Facility at CSIR-Central Drug Research Institute (CDRI), Lucknow, India and fed on standard pellet diet and water *ad libitum*. The animals and the animal experimental procedures were approved by the Animal Ethics Committee of CDRI duly constituted under the provisions of CPCSEA (Committee for the Purpose of Control and Supervision on Experiments on Animals), Government of India. The study bears the approval no. 129/08/Para/IAEC/renew (84/09) dated 27. 04. 2009.

### Parasite isolation

Adult male and female *B. malayi* worms were collected from the peritoneal cavities of the infected jirds on day 80–85 post larval inoculation. The worms were washed in culture medium RPMI-1640 containing 2 mM L-glutamine, 25 mM HEPES, 100 U/ml penicillin, 100 mg/ml streptomycin and 2.5 mg/ml amphotericin B. The individual worm was placed in 1 ml of the culture medium in a 48 well culture plate and kept at 37°C under 5% CO_2_ in air for 2 h for acclimatization. The release of microfilariae (Mf) in culture medium was assessed and the viable fertile female worms were selected for RNAi treatment. The infective larvae (L3) of *B. malayi* were isolated from the infected *Aedes aegypti* mosquitoes [15], [16] and washed several times in the fresh culture medium. The mature and highly motile L3 were selected for siRNA treatment.

### siRNA treatment of *B. malayi* adult worms by soaking method

The RNAi studies were carried out in the adult worms of both sexes by the soaking method. The culture medium and negative siRNA served as the controls in all the experiments. The adult parasites (4 female +2 male worms) were kept in Geba Flex dialysis tube (5 kDa cut off) containing 1 mM spermidine, 8 U RNAse OUT and 5 µM of siRNA in 800 µl medium. Six such tubes were kept in a beaker containing 300 ml of RPMI medium preheated to 37°C. The beaker was incubated at 37°C in a CO_2_ incubator for 60 h. At first 12 h, one tube was removed from the beaker and the adult worms were transferred to the fresh medium. The left over medium in the tube was centrifuged at 800 g for 2 min to pellet the contents which were suspended in 50 µl medium and microscopically examined to assess the number and phenotype of the *in vitro* released Mf. Of these four female worms, two were frozen in the Trizol reagent for preparation of nucleic acid to measure mRNA expression of *tpp* by real time PCR (qRT-PCR). The remaining two females and 2 males were transferred to the fresh pre-heated culture medium (37°C) for 30 min and their motility was assessed by scoring and the worm viability was subsequently checked by MTT reduction assay using the dye 3-(4,5 dimethylthiazol-2-yl)-2,5 diphenyl tetrazolium bromide. The remaining three tubes were removed after 24 h, 36 h and 48 h and handled in the same way. The two left over tubes were later removed at 60 h of incubation. Of the 8 female worms obtained from these two tubes, two were frozen in the Trizol reagent for qRT-PCR while remaining 6 female and 4 male worms were transferred to fresh siRNA free medium. These worms were incubated for another 48 h by replacing the medium with fresh normal medium at every 24 h. At the end of the experiment i.e. 48 h after transfer to the siRNA free medium, 2 out of 6 females were teased to microscopically observe intrauterine contents to determine the effects of silencing on embryogenesis as revealed by the presence of relative proportions of various progenies. The other 2 females were frozen in Trizol reagent for qRT-PCR while the remaining 2 females and 4 male worms were checked for their motility and viability and *in vitro* Mf release in the culture medium as discussed above. Three experiments were carried out with the same number of worms under identical conditions and the data are expressed as mean ± SD of the three experiments.

### Quantification of microfilariae release and their phenotype

The Mf pelleted at various time points were suspended in 50 µl of PBS as mentioned above and 10 µl of this suspension in triplicate was used for assessing the number of Mf released *in vitro*. Mf suspension was made in to a thin smear on glass slide which was later fixed and stained with Giemsa to observe the phenotypic changes and the photographs were taken by a colour digital camera (Nikon, Japan).

### Motility scoring and viability of *B. malayi* adult worms

The viability of adult worms was assessed by the mitochondrial reduction of 3-[4,5-dimethylthiazol-2-yl]-2,5-diphenyl tetrazolium bromide (MTT; Sigma) to formazan as described earlier [16], [17]. The formazan formed was quantified spectrophotometrically at 530 nm in a multiplate reader (Tecan, Infinite M-200, Switzerland). The motility scoring of the adult worms was carried out as, 0% motility reduction = 5; 1 to 25% = 4; 26 to 49% = 3; 50 to 74% = 2; 75 to 99% = 1 and 100% as dead. The loss in motility of adult worms and the percentage inhibition in MTT reduction in treated adult parasites were compared with that of respective untreated controls.

### Measurement of mRNA expression of *tpp* by real-time PCR (qRT-PCR)

The loss of specific transcripts following RNAi treatment was examined by real-time quantitative RT-PCR (qRT-PCR). *B. malayi* β-tubulin gene (Bm-tub-1) was used as an endogenous control gene. Glyceraldehyde-3-phosphate dehydrogenase mRNA levels were also checked in the treated and control worms to determine specificity of the RNAi. For qRT-PCR, the primers were designed with Beacon designer software (Table 1). The frozen worms were homogenized in Trizol reagent and the RNA was extracted as described earlier [18]. In brief, the first strand cDNA was generated using the Super Script III first strand cDNA synthesis kit (Invitrogen, USA) using oligo (dT) 20 primers. The specific cDNA fragments were then amplified by real-time PCR using SYBR premix and Roche applied System (Roche, US). The PCR conditions for qRT-PCR were 95°C for 5 min, followed by 40 cycles of 95°C for 20 s, 56°C for 15 s, and 72°C for 30 s. Relative amount of target amplicon in each experiment was determined by comparative ΔC_T_ method. The value of the control group (siRNA free medium) was set to 100% and the relative mRNA levels were expressed for each group.

**Table 1:** Primer sequences used in real-time RT-PCR (qRT-PCR) with the respective gene accession numbers.

Target Gene	Primer name	Primers (5′-3′)	Gene Accession number
Bm-tpp	Bm-tpp F	5′AAGATGGTATTACGGATG3′	Bm1_08695
	Bm-tpp R	5′TGTTGGATAATTCAGAAGT3′	
Bm-tub-1	Bm-tub F	5′ ACT TGG TGT CCG AAT ATC3′	Bm1_25780
	Bm-tub R	5′ACTCTTCCTGTTCAATGTAT3′	
Bm-G6PD	Bm-G6PD F	5′GGA GCG GAG TAT GTT GTG3′	Bm1_41940
	Bm-G6PD R	5′CAT TAG AGA TGA TAT GAT TGT TGG3′	

### Silencing of the *tpp* gene in L3

Two hundred *B. malayi* L3 were cultured in the 48 well plate containing 1 ml culture medium fortified with 1 mM spermidine, 8 U RNAse OUT and 2 µM siRNA. The medium and the negative siRNA controls were set up in parallel. The plates were incubated at 37°C, in 5% CO_2_ for 24–48 h and the motility of larvae was scored after 30 min of transfer to the fresh siRNA free medium. The L3 were frozen in the Trizol reagent for the measurement of the *tpp* transcript level. To further examine the effects of *in vitro* RNAi treatment on *in vivo* development of L3 in the susceptible rodent host- jirds, more sets of L3 were treated with the siRNA in the same way for only 24 h. From this culture, actively motile L3 were selected, washed and 100 L3 were inoculated into the peritoneal cavity of a 6 week old, male jird. A total of 3 jirds/group could be inoculated with 100 L3 each in this way. On day 120 post inoculation when the L3 get established as the sexually mature adult parasites, the jirds were euthanized and the worms from the peritoneal cavity were isolated by peritoneal washing and counted. The worms were measured and the females were teased in a drop of PBS to observe intrauterine development.

### Statistical analysis

Data were analyzed using one-way and two-way (for grouped data) analysis of variance (ANOVA) with the help of statistical software PRISM 5. Individual comparisons following ANOVA were made using the Bonferroni method. The criterion of evaluating statistical significance between the experimental and control groups was as follows: *p* value<0.05 was considered significant and marked as *, *p*<0.01 as highly significant and marked as **, *p*<0.001 was very highly significant and marked as ***.

## Results

### Silencing of tpp gene transcript results in to impaired viability of adult *B. malayi*

The siRNA mediated *tpp* gene silencing reduced the viability of both male and female worms, though, the parasites remained alive till the end of experiment (Table 2). There was ≥50% inhibition in reduction of MTT within 24 h of soaking in *tpp*-specific siRNA and this reduction amplified as the duration of exposure increased reaching maximum (75–80%) at 48 h of treatment.

**Table 2:** Effect of *tpp* gene silencing on viability of both adult male and female parasites of *B. malayi* was assessed by motility scoring and MTT reduction assay.

	Motility score (female worms)			% Inhibition in MTT reduction		Motility score (male worms)			% Inhibition in MTT reduction	
Time	Control	Negative siRNA	Gene specific siRNA	Negative siRNA	Gene specific siRNA	Control	Negative siRNA	Gene specific siRNA	Negative siRNA	Gene specific siRNA
12	5	5	3	6.03±4.1	48.52±7.8	5	5	3	6.16±5.8	48.16±8.2
24	5	5	3	5.6±1.6	49.9±11.1	5	5	2	6.8±4.3	60.5±8.1
36	4	5	2	6.16±1.6	63.8±13.1	4	4	2	6.6±3.9	64.83±8.5
48	4	4	2	6.66±1.0	73.92±2.6	4	4	2	9.3±5.9	74.5±3.2
108 h	4	4	2	6.9±2.2	78.18±5.1	4	4	1	11.33±9.6	87.33±6.2

5 = 0% motility reduction, 1 to 25% reduction = 4, 26 to 49% reduction = 3, 50 to 74% reduction = 2; 75 to 99% reduction = 1, 100% = Death.

### *In vitro tpp* gene silencing caused reduced release of Mf, their viability and altered phenotype

**Figure 1:**
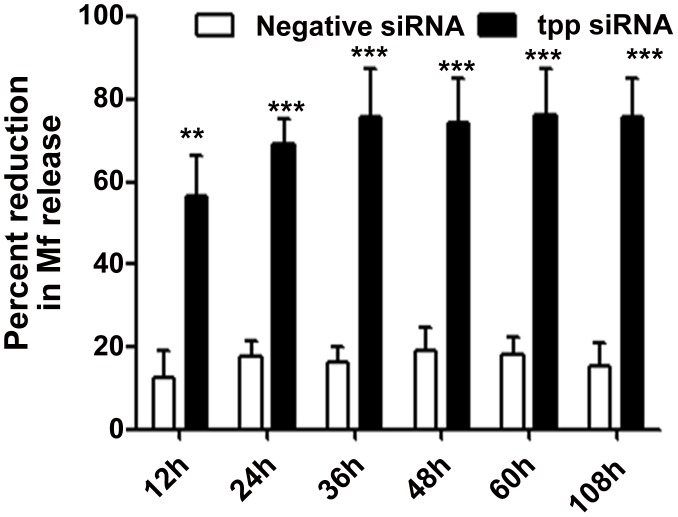
Treatment of adult female *B. malayi* with *tpp* siRNA leads to an inhibition of *B. malayi* microfilaria release *in vitro*. Geba flex dialysis tubes containing 4 worms each were treated with 5 µM siRNA for different time periods (12, 24, 36, 48, 60 h). 6 females after 60 h treatment were cultured for a further two days in normal culture medium (108 h). Mf release was recorded at these different times and expressed as a reduction in release in comparison to the respective siRNA free medium control group. The criterion for statistical significance between the results was *p* value<0.05 was considered significant marked as *, <0.01 as highly significant**, <0.001 *** as very highly significant.

**Figure 2:**
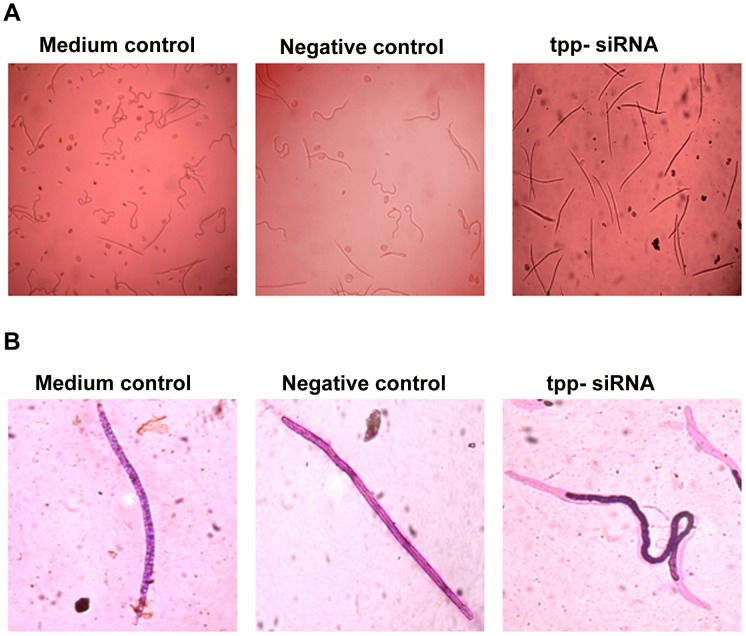
*tpp* silencing induced death and phenotypic abnormalities in microfilariae. (A) Treatment of adult female *B. malayi* with *tpp* siRNA lead to death of majority released microfilariae. Larval lethality was noticed after 12 h of treatment and persists till the end of the experiment. (B) Apart from death microfilariae released by t*pp* siRNA group also showed phenotypic abnormalities, i.e. their body is contracted leaving one or both side of sheath empty. Microfilariae released by controls and siRNA treated female worms were stained with Giemsa stain and visualized through microscope and pictures were captured with Nikon colour digital camera.

The Mf released from the two controls and siRNA treated female parasites in culture medium were counted. A considerable reduction in the number of Mf released by the females in presence of *tpp* specific siRNA was noticed within 12 h of the treatment which reduced further (∼74%) at 48 h. This effect persisted even after transferring the worms to siRNA-free medium for another 48 h (total 108 h). The reduction in Mf release was specific for Bm-tpp siRNA since no significant reduction in negative control worms was noticed (*p*<0.001) (Figure 1). tpp-siRNA treatment also resulted in to the death of ∼90% of the released Mf within 12 h of exposure while remaining 10% got paralyzed and seldom revealed slight movement of the anterior or posterior ends displaying gene silencing effects (Figure 2A). The Mf exhibited “shriveled phenotype” leaving the inside of sheath empty showing signs of acute structural deformities (Figure 2B).

### The *tpp* gene silencing adversely affected the embryogenesis of female worm

**Figure 3:**
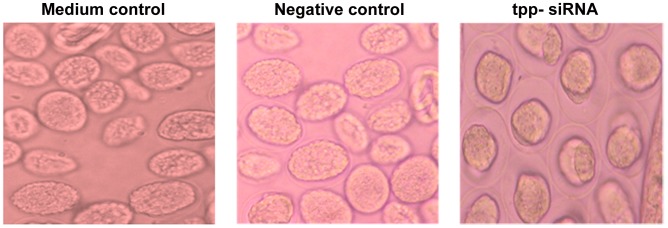
Treatment of adult female *B. malayi* with *tpp* siRNA leads to phenotypic changes in developing embryos. Intrauterine progeny from individual female worms were examined 2 d after treatment with medium control, negative control, and *tpp* siRNA. The eggs from *tpp* siRNA treated female worms were degenerated showing increased space between egg shell and embryo.

**Figure 4:**
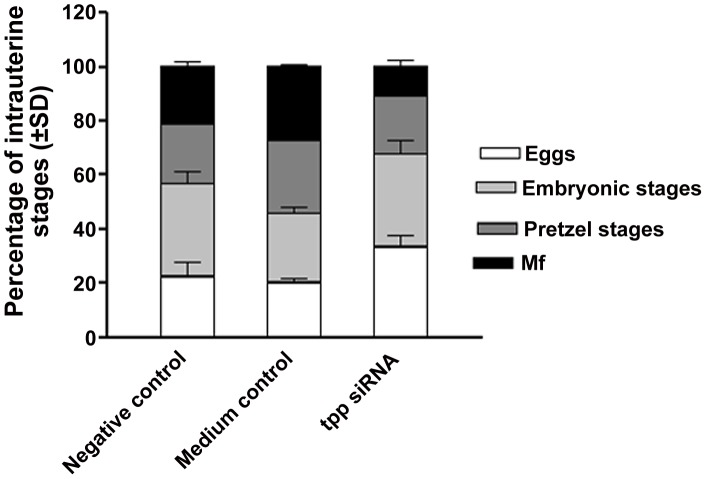
Effect of *tpp* siRNA treatment on adult female worm embryogenesis. Intrauterine progeny from individual female worms (groups of four adult worms) were examined two day after siRNA treatment and expressed as the relative proportions of progeny at different stages of development.

The intrauterine contents of female worms incubated in presence of *tpp* specific siRNA showed all degenerated eggs indicating developmental arrest at an early developmental stage. The eggs appeared granulated leaving a big space inside the eggshell (Figure 3). The percentages of pretzel stage and hatched Mf inside the uteri decreased tremendously (*p*<0.01) while the early stages of eggs increased marginally when the worms were teased at the end of incubation i.e. 108 h (Figure 4).

### siRNA treatment of adult *B. malayi* leads to loss of *tpp* transcript

**Figure 5:**
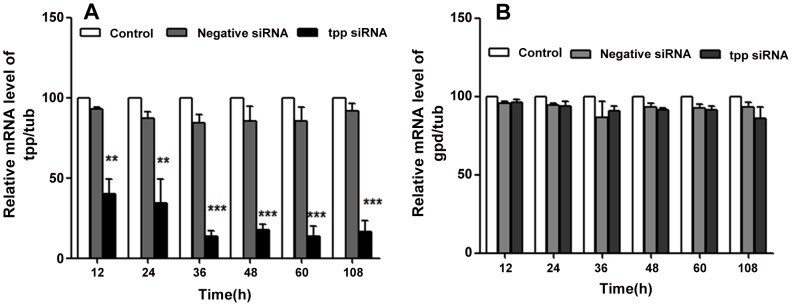
Soaking *B. malayi* adult female worms in siRNA results in Bm-*tpp* gene-specific inhibition of expression. Quantative Real time PCR. Two female worms at different time interval (12, 24, 36, 48, 60 h) and 2 day after treatment were removed and analyzed for difference in *tpp* and *gpd* gene-specific transcript levels by qRT-PCR. The relative amounts of *tpp* and gpd amplicon were determined by using the comparative ΔCT method and normalizing against the endogenous control gene (Bm-tub-1). The median value of the control group was set to 100% and the percent relative mRNA level was shown. (A) Soaking *B. malayi* adult female worms in siRNA results in *tpp* gene-specific inhibition of expression. (B) siRNA treatment of adult worms non significantly effects the expression level of gpd mRNA. The criterion for statistical significance between the results was *p* value<0.05 was considered significant marked as *, <0.01 as highly significant**, <0.001 *** as very highly significant.

The analysis by qRT-PCR on RNA isolated from the RNAi treated worms showed ∼60% reduction in the *tpp* gene transcript level within 12 h of treatment. The reduced transcription reached up to 86% and this level stayed till the end of the experiment (60 h) even after 48 h of transfer of worms to siRNA free medium. The reduction in transcript level was highly significant (*p*<0.001) when compared with that of negative control which demonstrated merely 6–15% of reduction. The reduction in GPD mRNA level was found to be between 2 and 12% in negative and tpp siRNA treated worms demonstrating a negligible off target effect on other genes. For data presentation, mRNA levels were normalized using β-tubulin (Bm-tub-1) transcript as a housekeeping gene control (Figure 5A and B). The reductions in Bm-tpp gene specific transcript level could be well correlated with the reduced release of Mf by female worms, death and phenotypic changes in the released Mf and adverse effects on the intrauterine development with changes in the percentage of different progenies.

### *tpp* gene silencing was lethal for L3 and those defied lethal effects had impaired *in vivo* development in jirds

**Figure 6:**
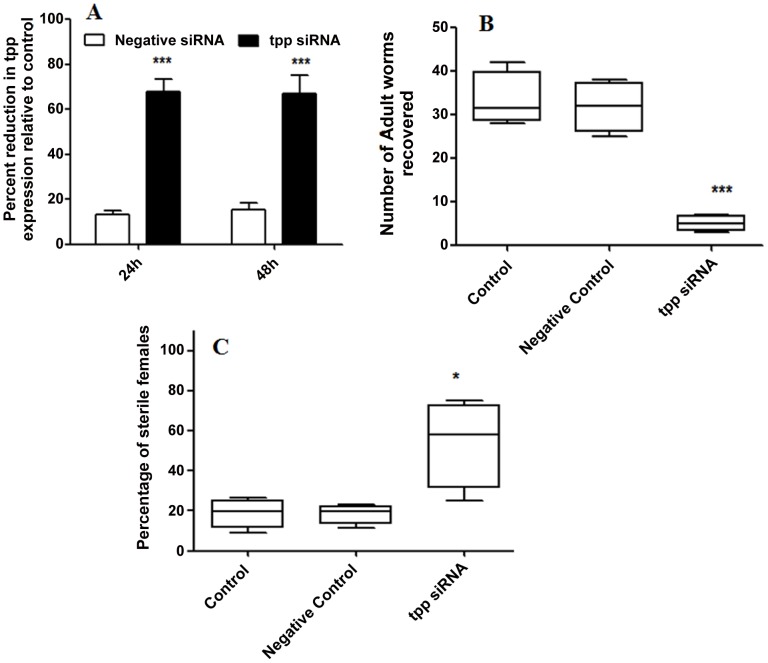
*In vitro* treatment of infective larvae with Bm-tpp siRNA results in to reduction in tpp gene expression and impairs their *in vivo* development in jirds. (A) siRNA treatment of infective larvae (L3) resulted in to significant loss of Bm-*tpp* transcript level after 24 and 48 h of incubation. (B) *In vitro* silencing of tpp gene in L3 reduced *in vivo* establishment of adult worm in jirds. L3 were cultured with *tpp* siRNA, negative siRNA and in medium for 24 h. After incubation ∼100 L3 were inoculated in the peritoneal cavity of jirds and animals were euthanized on 120 day post inoculation for recovery of adult worms. (C) Significant number of female worms recovered from siRNA treated group displayed defective embryogenesis. All female were recovered from animals were teased in PBS on glass slide. Condition of intrauterine contents (eggs, embryonic stages pre-Mf, Mf) was analyzed microscopically (Nikon). Percent of sterile females was calculated with respect to total female worm recovered and analysed. The criterion for statistical significance between the results was *p* value<0.05 was considered significant marked as *, <0.01 as highly significant**, <0.001 *** as very highly significant.

The silencing of *tpp* gene in L3 brought about lethality of majority of the larvae within 48 h. Within 24 h, 15% L3 showed mortality as opposed to 7–10% in controls (Table 3). qRT-PCR of larvae at 24 and 48 h post treatment revealed 70% reduction in the *tpp* transcript level (*p*<0.001) (Figure 6A). Like adult worms, GPD levels after siRNA treatment did not show any noticeable change (data not shown). The actively motile L3 which defy death after 24 h of incubation in *tpp* siRNA, siRNA free or negative control, were inoculated in to the peritoneal cavity of jirds to observe adverse effects of *tpp* silencing on their further *in vivo* development and establishment as adult worms. There was 84.9% reduction in the worm establishment over that of negative control where only 4% reduction was noticed (*p*<0.001). A significant proportion of the recovered female worms (54.5±22.03%) (*p*<0.05) had defective embryogenesis (Figure 6B and C). However, no significant difference in the lengths of the recovered adult parasites was seen in presence or absence of specific or off-target siRNA (Table 4).

**Table 3:** Effect of *tpp* siRNA treatment on morphological state of Infective larvae (L3).

Infective Larvae condition	Medium Control		Negative control		*tpp* specific siRNA	
	24 h	48 h	24 h	48 h	24 h	48 h
Active (%)	82.5±4.9	58.5±12.0	82.5±7.7	57.5±9.1	78.5±20.5	10.5±3.5
Sluggish (%)	10±2.8	17.5±3.5	12.5±3.5	17±5.6	9.5±7.7	22±1.4
Dead (%)	7.5±2.2	24±8.4	10±2.8	25.5±3.5	12±12.7	67.5±4.9

**Table 4:** Measurement of male and female worms length recovered from jirds infected with siRNA treated (*tpp* specific, negative) and untreated infective larvae.

	Worm length (cm±S.D.)	
Groups	Female worm	Male worm
Control	3.47±0.57	1.67±0.34
Negative siRNA	3.48±0.65	1.75±0.25
*tpp* specific siRNA	3.51±0.71	1.68±0.29

## Discussion

Trehalose-6-phosphate phosphatase (TPP) enzyme is involved in the biosynthesis of the trehalose where it dephosphorylates trehalsoe-6-phosphate to yield trehalose. TPP belongs to the HAD (L-2-haloacid dehalogenase) super family of magnesium-dependent phosphatases/phosphotransferases and is present in both prokaryotes and eukaryotes [17], [19], [20]. We have recently reported that *B. malayi* TPP shows magnesium dependent unusual phosphatase activity and it is expressed by all the three major life-stages of parasite [13]. In the present study, the biological functions of *B. malayi* TPP employing have been investigated by employing RNAi technique using small size siRNA which validated it to be an antifilarial drug target. RNAi is a powerful technique to address both target validation and inhibitor specificity in the drug discovery. It has been successfully applied to various nematode species [21]–[23] including some filarial species such as *Onchocerca volvulus* [24], [25], *Litomosoides sigmodontis* [26] and *B. malayi* [25], [27], [28]. Aboobaker and Blaxter (2003) were the first to show the successful knockdown of three *B. malayi* genes; beta tubulin, RNA polymerase II large subunit and Mf sheath protein 1 by 300 bp dsRNA. However, the use of short length siRNA proved to be a more efficient method of silencing any target gene [18], [24], [29]. In the current investigation, *tpp* specific siRNA of 21 bp has been used to silence *tpp* gene in various life cycle stages of *B. malayi*. In *B. malayi rde-4, rde-2, sid-1, sid-2* and *rsd-2* genes have not been reported so far that are known to be responsible for uptake of dsRNA in *C. elegans* [22]. Successful dsRNA uptake and subsequent gene silencing in absence of these genes in *B. malayi* indicates the involvement of some other genes or divergence of these genes during evolution in the parasites. The *rsd-3* gene of *C. elegans* is involved in the systemic RNAi of exogenous dsRNA. A homologue of this gene has been identified in *B. malayi* genome [30], [31] indicating the possible role of *rsd-3* in making filariids more vulnerable to RNAi. Concentration of siRNA also matters for a successful gene silencing where high concentration may induce stress response and low levels can be inefficient silencers. A concentration of 5 µM of siRNA has earlier been shown (including our own study) to be efficient in silencing genes in filarial parasites, *B. malayi, Onchocerca volvulus* without inducing any off target effects [18], [24], [27] and this concentration could also efficiently silence the *tpp* gene in the current investigation.

The loss of *tpp* function in *B. malayi* led to a drastic reduction in the Mf release from the adult female *B. malayi* and ∼90% of the released Mf were found dead. A large proportion of the released Mf revealed severe phenotypic deformities such as contraction of the body on on one or both sides leaving a long empty space at both ends. The embryograms of the treated female worms displayed the presence of a large number of degenerated eggs and arrested embryogenesis at an early embryonic development stage. The reduced motility of adult male and female worms together with impaired viability was noticed with in 12 h of soaking in specific siRNA indicating diminished or impaired synthesis of trehalose. In the nematodes, the trehalose is known to act as a stress protectant, energy source, assist in glucose uptake and egg hatching. However, its role as stress protectant in filarial parasite still seems uncertain because filariids are the tissue dwelling nematodes that rarely face desiccation, freezing or osmotic stress, however, it may serve as an energy source in filarial parasites in the same manner as in *Ascaris suum* [32], [33] where it is present in the muscles, reproductive system and hemolymph with utmost content in the reproductive system. The TPS and TPP enzyme activity is high in uterus containing fertilized eggs suggesting an imperative role of trehalose in embryogenesis. Passey and Fairbairn (1957) also suggested trehalose to be the main source of energy during an initial phase of egg development in *A. suum*. The embryo development in *B. malayi* follows a definite pattern as in other nematodes and siRNA mediated *tpp* silencing greatly impaired the progression of early embryonic development that resulted in to the degeneration of existing eggs and further embryonic development arrest. In Arabidopsis AtTPS1 enzyme plays a major role in the cell division and cellular metabolism during embryo development and gene mutation causes lethal embryonic phenotype [34]. The *tpp* gene silencing could also have promoted a high concentration of intermediate T-6-P due to the loss of TPP activity as observed in *C. elegans* where mutation in *gob-1* gene encoding TPP leads to an early larval lethality [13]. The lethal effects of trehalose-6-phosphate were also demonstrated in *O. volvulus*, where it has been shown to be a better micro- and macrofilaricidal agent as compared to diethylcarbamazine. The inhibitory action of T-6-P on enzyme trehalase was postulated as an action mechanism of trehalose-6-phosphate [35]. In the present study, the toxic build up of T-6-P might have affected the sugar metabolism of *B. malayi* either via hexokinase as reported for *Saccharomyces cerevisiae* [36] or trehalase enzymes causing reduced survival of *B. malayi*, larval lethality and phenotypic abnormalities.

A majority of the *B. malayi* L3 exposed to *in vitro tpp* gene silencing were found dead. The L3s which survived and defied any visible adverse effect of siRNA, were inoculated in to the peritoneal cavity of naïve jirds to investigate their further *in vivo* development up to the adult stage and subsequent survival. The jirds were euthanized to recover the adult parasites and observe the female reproductive potential by observing the intrauterine development, the production and release of Mf by the parasites was also documented after euthanizing them at the patent stage of infection when the parasites have already reached sexual maturity and fertile females produce and release large number of Mf in the peritoneal cavity. It was observed that a majority of the *tpp*-specific siRNA treated L3 died and did not develop in to adult stage demonstrating major reduction in the establishment of adult worms. Not only this, a substantial proportion of the recovered female worms displayed defective embryogenesis. These *in vivo* studies further confirm that the disruption of the trehalose biosynthetic pathway by silencing the *tpp* gene can adversely affect the establishment and further development of *B. malayi* in the vertebrate host. Such effects have already been demonstrated in a variety of plant and human parasitic pathogens where disruption of trehalose biosynthetic enzyme affected the infectivity of pathogens [37]–[41]. Ours is the first report on the effect of any gene silencing on *in vivo* development of any filarial sp. Trehalose has also been shown to act as a free radical scavenger molecule in protecting proteins and membranes from oxidative damage [42], [43]. In case of *B. malayi* L3, the lack of trehalose due to disruption of its biosynthesis possibly rendered L3 vulnerable to oxidative attack imposed by the jirds' peritoneal immune cells. The present findings also correlated well with our vaccination studies where recombinant TPP provided substantial (67.8%) protection against *B. malayi* larval challenge signifying the important role of trehalose and its enzymes in parasite establishment (Unpublished data). In the present study, the gene silencing resulted in to the specific reduction in *tpp* mRNA levels. The immunodetection of TPP or enzymatic activity of TPP in the lysate of siRNA treated worms may further define whether the knockdown of tpp mRNA translates in to specific reduction in TPP enzyme levels or activity.

The off target effects of RNAi treatment have been demonstrated in the filarial nematodes where the control dsRNA was found to induce several phenotypic abnormalities along with the nonspecific changes in the mRNA levels [23]–[26]. In mammalian systems, dsRNA longer than 30 bp activates interferon-γ response pathway leading to general shutdown of protein synthesis and apoptosis. 21 base pair duplexes with two nucleotide 3′ overhangs circumvent stress response [44], [45]. The 21 base pair size of negative siRNA in the present study demonstrated negligible off target effects on the parasites as well as mRNA expression of tpp gene. The siRNA treatment of adult worms also did not induce generalized silencing effects in treated worms thus further circumventing the possibility of any off target effects.

The present findings demonstrate the essential and indispensable role of trehalose-6-phosphate phosphatase enzyme in filarial parasite fertility, development and survival. The trehalose pathway has been much less explored in parasitic nematodes and majority of the findings are derived from the free living nematodes. Further investigation on the inhibition of hexokinase activity by T-6-P is currently underway in our lab and the findings would give an insight in understanding the biochemical mechanism of regulation of sugar metabolism in *B. malayi*.
